# Evaluation Study of Ammonium Removal from Groundwater by Electrodialysis: Case Study of Real Groundwater from the City of Kenitra in Morocco

**DOI:** 10.1002/open.202300163

**Published:** 2024-04-09

**Authors:** Mohamed Hazra, Mohamed Ouzbair, Ibrahim Maolida, Omar Elrhaouat, Mustapha Tahaikt, Azzedine Elmidaoui, Mohamed Taky, Sakina Belhamidi

**Affiliations:** ^1^ Chemistry Superior School of Technology Ibn Tofail University P.O. Box 1246 Kenitra Morocco; ^2^ Chemistry Laboratory of Advanced Materials and Processes Engineering Faculty of Sciences Ibn Tofail University P.O. Box 1246 Kenitra Morocco; ^3^ Biology Laboratory of Natural Resource and Sustainable Development, Faculty of Science P.O. Box 1246 Kenitra Morocco

**Keywords:** Demineralization rate, Electrodialysis, Removal ammonium, Water treatment, Water shortage

## Abstract

In this work, we investigated the feasibility of ammonium removal by electrodialysis (ED), a well‐known electro‐membrane process, based on the selective migration of anions and cations through anion exchange membranes (AEMs) and cation exchange membranes (CEMs). ED experiments are performed using a laboratory pilot. The ion exchange membranes (IEMs) pair used is AXE/CMX, AXE as an AEMs and CMX as a CEMs. The first tests are performed with real groundwater solutions from Kenitra city (Morocco), spiked with an initial concentration of 3 mg/L NH_4_Cl. The results gave a specific demineralization (SD) to NH_4_^+^ ions of 84.60 %; for a demineralization rate (DR) of 80 % and a time of 60 min. The multivariate principal component analysis (PCA) presents a total inertia of 99.23 %, the majority of the variables of which are positively correlated on the C1 axis with a variance of 95.5 % than that of C2 of 3.78 %. The quality of the diluted water determined by the Legrand‐Poirier method showed that the water was aggressive and that the addition of 4.54 mg/L Ca^2+^ was necessary to balance the water and make it fit for human consumption.

## Graphical abstract

**Ammonium in water**. Consumption of water polluted by ammonium (NH_4_^+^) can cause serious illnesses such as methaemoglobinaemia, mainly in children, as well as cancer. In addition, high concentrations can lead to eutrophication of aquatic environments. The electrodialysis process used achieved a good percentage of ammonium removal from the groundwater studied.

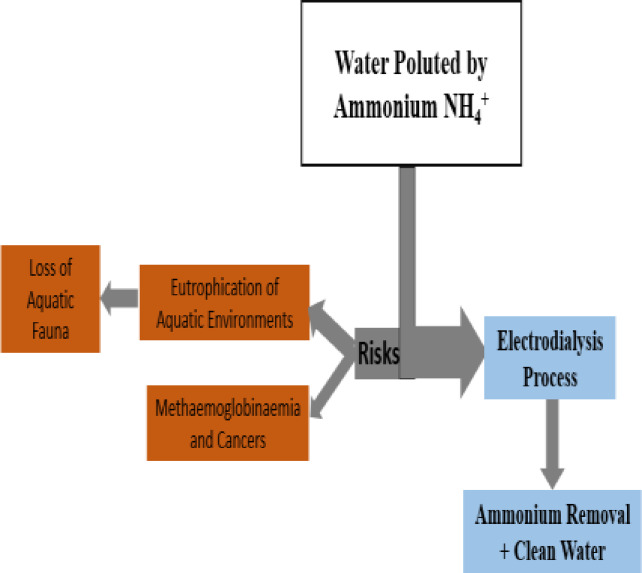

## Introduction

In Morocco, the agricultural sector occupies an important socio‐economic position; however, the evolution of farming practices is closely linked to the substantial demand for water resources needed to irrigate farmland. Previous studies have highlighted the excessive importance attached to intensive farming techniques and productivist models, which often neglect the conservation of natural resources.^[1]^ Examples include the Ziz basin in south‐eastern Morocco^[2]^ and the Souss‐Massa basin in central‐western Morocco,^[3]^ both of which are facing increasing pressure from excessive groundwater use and investment. What's more, the country‘s water scarcity crisis is set to intensify, with an anticipated 80 % depletion of water resources in the coming decades, mainly due to reduced rainfall associated with global warming and overexploitation of groundwater.^[4]^

These problems are exacerbated by the widespread use of chemical fertilizers, particularly nitrogen‐based, in agriculture, which results in significant contamination of water resources surrounding agricultural areas. In addition, the discharge of insufficiently treated wastewater is another source of pollution of water resources. It is imperative that Morocco effectively treats its wastewater before discharging it into the natural environment, to avoid further contamination of dwindling water resources and to mitigate the worsening water scarcity crisis.^[5]^ Ammonium pollution of water resources is caused by agricultural nitrogen fertilizers and wastewater discharges. Studies carried out in various regions of Morocco to assess groundwater and surface water quality have revealed severe ammonium pollution, exceeding the maximum permitted standard of 0.5 mg/L.[^6^, ^7^, ^8^, ^9^]

Ammonium (NH_4_^+^) is the acid conjugate of the acid‐base couple (NH_4_^+^/NH_3_), the gaseous form ammonia (NH_3_) being more toxic than its ionized counterpart, ammonium (NH_4_^+^). In aqueous media, the two forms coexist in equilibrium, with one prevailing as a function of pH and water temperature. The known effects of these compounds on human health and the environment are significant; their transformation into nitrites and then nitrates by nitrifying bacteria constitutes a risk. Consumption of nitrate‐contaminated water can lead to methaemoglobinaemia, particularly in infants under six months of age,^[10]^ and is suspected of contributing to certain forms of cancer.^[11]^ Ammonium thus contributes indirectly to the development of methaemoglobinaemia and certain cancers, including colorectal cancer, thyroid disease and neural tube defects. In aquatic environments, high levels of ammonium promote eutrophication, leading to the disappearance of various aquatic species, including fish.^[12]^

The use of membrane processes, notably electrodialysis (ED), to treat water containing high levels of contaminants is widely recognized.^[13]^ ED works by removing or concentrating ionic compounds from aqueous solutions through ion exchange membranes (IEMs) under the influence of an electric field. A ED cell typically comprises alternating anion exchange membranes (IEMs) and cation exchange membranes (CEMs) separated by spacers, with electrodes located at the end of the modules. The elementary cell consists of two compartments: the diluted compartment and the concentrated compartment. By supplying an electrolyte solution AX (A^+^, X^−^), an electric field causes anions and cations to be depleted in the dilute compartment and enriched in the concentrate compartment.

Compared with other membrane methods such as reverse osmosis, ED is considered promising due to its extended service life, its ability to operate at elevated EMIs temperatures, and its reduced susceptibility to membrane fouling and scaling. It is considered economically viable and most efficient when feed stream salinity is below 5 g/L.[^14^, ^15^] ED operation depends on the development of IEMs that facilitate high water recovery without the need for phase changes, chemical reactions or additives. These advantages benefit the environment by reducing dependence on fossil fuels and chemical detergents. Several studies have demonstrated the effectiveness of ED in removing ammonium from wastewater.[^16^, ^17^, ^18^] In particular, ammonium can be recovered from the concentrate compartment and recycled as a nutrient. For example, Xia et al^[19]^ used ED to improve the efficiency of hydrogen production from wastewater by hydrogen‐producing bacteria through ammonium removal. In addition, unlike other processes, ED enables the concentrate stream to be reused as fertilizer for plant fertigation, with the aim of achieving zero liquid discharge (ZLD) to promote the circular economy and sustainable development.^[20]^ However, research on ED has primarily concentrated on the removal of contaminants from wastewater, with limited exploration of real groundwater treatment, partly due to the complex chemistry of water.^[13]^ One of the key advantages of ED is its capability to remove a wide range of ionic compounds from water. Nevertheless, excessive demineralization of water can render it aggressive for human consumption. Therefore, achieving balanced water at the conclusion of the treatment process is crucial.

The Legrand‐Poirier method is a well‐established and widely utilized approach for assessing water quality. This method involves representing water quality using a curve known as the calco‐carbonic equilibrium curve of water. The representation plane comprises two axes: the Ca^2+^ concentration on the abscissa and the total mineral carbon (TMC) or total CO_2_ concentration on the ordinate. The equilibrium curve divides the plane into two regions: aggressive water and scaling water. Through computer calculations of the calco‐carbonic equilibrium conditions and graphical representations, the evolution of water under the influence of treatments can be visualized.^[21]^

The aim of this study is to assess the feasibility of removing ammonium from groundwater using ED with AXE membranes as anion exchange membranes (AEMs) and CMX membranes as cation exchange membranes (CEMs). The treated water is intended for human consumption, with the aim of mitigating the loss of water resources compromised by high levels of pollution. As the natural concentration of ammonium in groundwater is generally lower than in wastewater, the initial ammonium concentration chosen for this study is based on previous assessments of the quality of ammonium‐polluted groundwater and surface water, as indicated in the references.

The quality of the water obtained (dilute) by ED will be assessed using the Legrand‐Poirier method, which evaluates water aggressiveness, scaling potential or equilibrium, as a function of the demineralization rate (DR). Statistical analysis, in particular Principal Component Analysis (PCA), is used to verify the results. PCA is a statistical technique used to explore and reduce the dimensionality of data, enabling a better understanding of relationships and patterns within the data set.

## Experimental Section

### Description of the ED Pilot Plant

**Figure 1:**
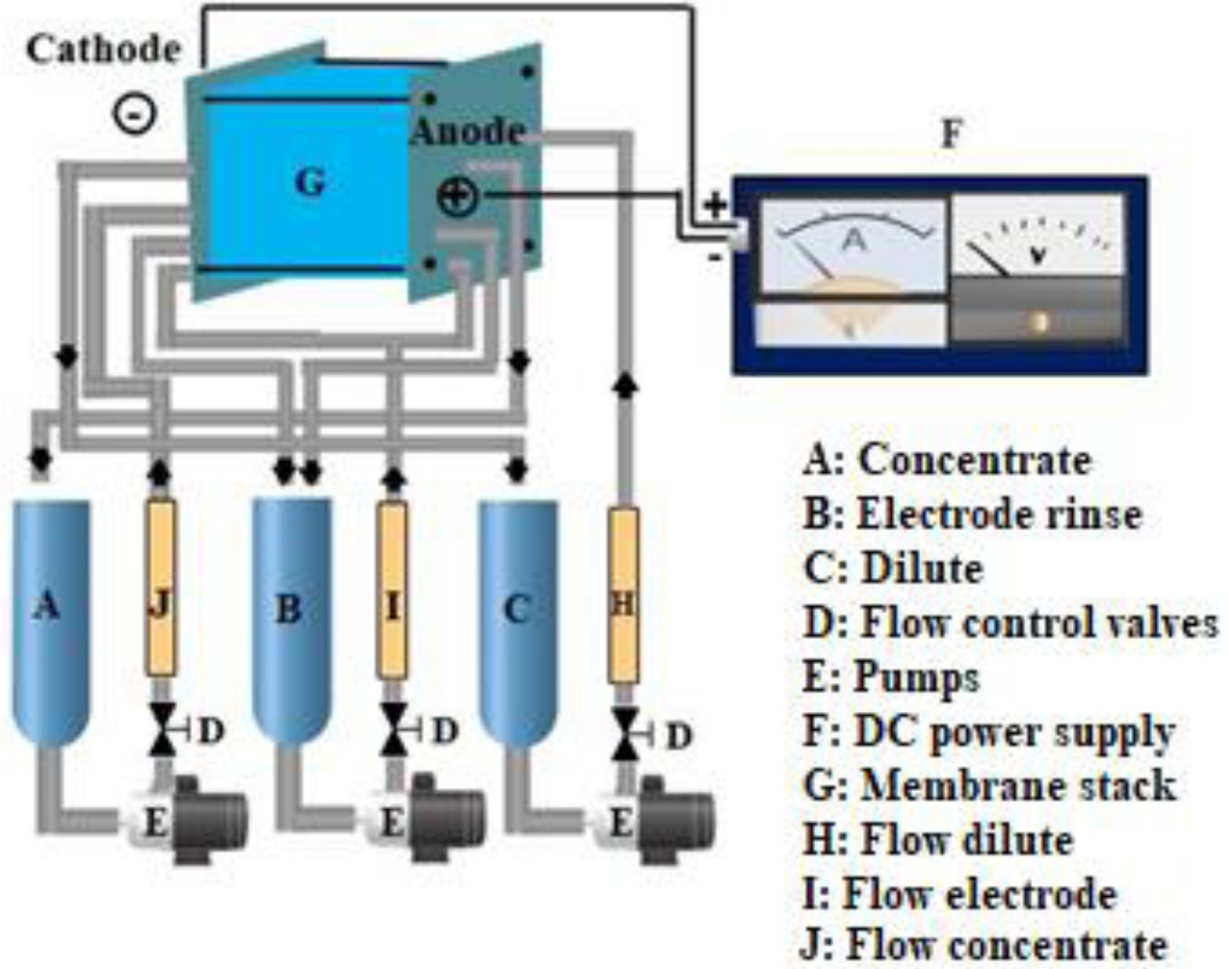
Schematic of the TS‐2‐10 electrodialyzer with the three tanks containing the dilute, concentrate and electrode rinse solutions, the pumps and the membrane stack.

**Figure 2:**
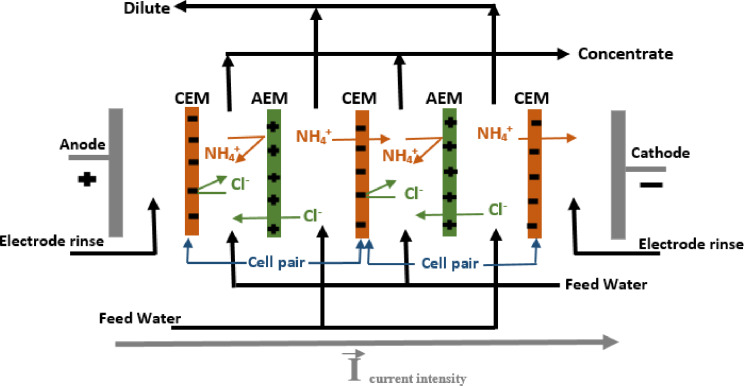
Selectivity of AXE and CMX membranes. **a)** SD of anions as a function of demineralization rate. **b)** SD of cations as a function of demineralization rate.

In this study, experiments were carried out using a ED pilot plant (TS‐2‐10) supplied by Eurodia Co (France), equipped with NEOSEPTA AXE membranes as anion exchange membranes (AEMs) and NEOSEPTA CMX membranes as cation exchange membranes (CEMs) manufactured by Tokuyama Co (Japan). A total of 22 membranes were used, including 12 CMX and 10 AXE. The experimental set‐up included three solution tanks, each with a capacity of 2 L, designed to hold dilute, concentrate and electrode rinse solutions. By applying a continuous electrical potential, ammonium ions migrate to the cathode, exit the dilute compartment, cross the cation exchange membrane and are blocked by the anion exchange membrane in the concentrate compartment. Schematic representations of the TS‐2‐10 pilot plant and the membrane stack exchange process are shown in Figures 1 and 2, respectively.

The characteristics of the TS‐2‐10 ED pilot plant used in this study correspond to those described in the work of Elmidaoui et al^[22]^ while the properties of the membranes used are detailed in the study by Addar et al.^[23]^

### Analyses Methods

The ED operations are performed with real groundwater solutions from Kenitra city (Morocco), spiked with an initial concentration of 3 mg/L NH_4_Cl. The use of real water allows us to control the actual effect of ammonium removal in the presence of other cations present in the water, enabling us to obtain results closer to reality than is often difficult to achieve when working with synthetic solutions. The characteristics of the feed water are presented Table 1.

**Table 1:** Characteristics of the feed water.

Parameters	Values
EC* (μS/cm)	686
T (°C)	19.6
pH	7.53
NH_4_^+^ (mg/L)	3.24
SO_4_^2−^ (mg/L)	36.47
NO_3_^−^ (mg/L)	22.48
P‐Alkalinity (meq/L)	0
T‐Alkalinity (meq/L)	4.7
Hardness (meq/L)	5.88
Ca^2+^ (mg/L)	103.33
Mg^2+^ (mg/L)	8.70
HCO_3_^−^ (mg/L)	286.58
K^+^ (mg/L)	2.06
Na^+^ (mg/L)	106.22

*EC: Electric conductivity

During the testing process, water samples were periodically extracted from the dilute stream, and ion concentrations were determined through analytical methods. A conductivity probe was immersed in the dilute water to continuously monitor conductivity throughout the process. At each demineralization rate reached (20 %, 40 %, 60 % and 80 %), water conductivity, time, and corresponding current intensity were recorded.

The alkali ions K^+^ and Na^+^ were measured spectrometrically using an industrial flame emission spectrometer model PFP7 JENWAY. NH_4_^+^ and SO_4_^2−^ ions were determined spectrometrically using a UV/VIS spectrometer model UV1600 at wavelengths of 655 nm and 650 nm, respectively. NO_3_^−^ ions were measured potentiometrically using selective electrodes and an ionometer model Hach SensionTM^+^ MM340. pH levels were measured using a Jenway 3510 pH meter electrode, and electric conductivity (EC) was measured using a WTW Inolab level 1 conductivity cell. Other parameters such as HCO_3_^−^, T‐Alkalinity, P‐Alkalinity, hardness, Ca^2+^, and Mg^2+^ were determined using standard methods as previously described.^[24]^ Other parameters such as time, demineralization rate (DR) and specific demineralization rate (SD) of each ion are also monitored. DR and SD are expressed by equations (1) and (2) respectively:

(1) $$DR\left(\%\right)=\frac{\left(E_{f}-E_{d}\right)}{E_{f}}\times 100$$

(2) $$SD\left(\%\right)=\frac{\left(C_{f}-C_{d}\right)}{C_{f}}\times 100$$

Where C_d_ is the solute concentration in dilute (mg/L) and C_f_ the solute concentration in the feed water (mg/L) of the ions considered.

The statistics carried out in this study are multivariate principal component analysis (PCA) using the jmp version 13 software.

## Results and Discussion

### Feasibility of Eliminating Ammonium Ions by ED

To assess the feasibility of ammonium removal via electrodialysis (ED), the following operating conditions were employed:

- The feed volume was standardized across all three compartments (dilute, concentrate, and electrodes).
- The dilute and concentrate compartments received identical raw solutions, while the electrode compartment was supplied with a solution consisting of water enriched with NaCl. This ensured that the electric conductivity of the electrode compartment remained higher than that of the dilute and concentrate compartments, thereby minimizing energy consumption.
- The flow rate was determined based on a prior study by Elmidaoui et al^[25]^ which optimized nitrate removal from groundwater using the same TS‐2‐10 pilot plant. The optimal flow rate identified in that study was 180 L/h.
- An applied voltage of 10 V was utilized.

These operating conditions were selected to evaluate the efficacy of ammonium removal from the initial solution via ED.

**Figure 3:**
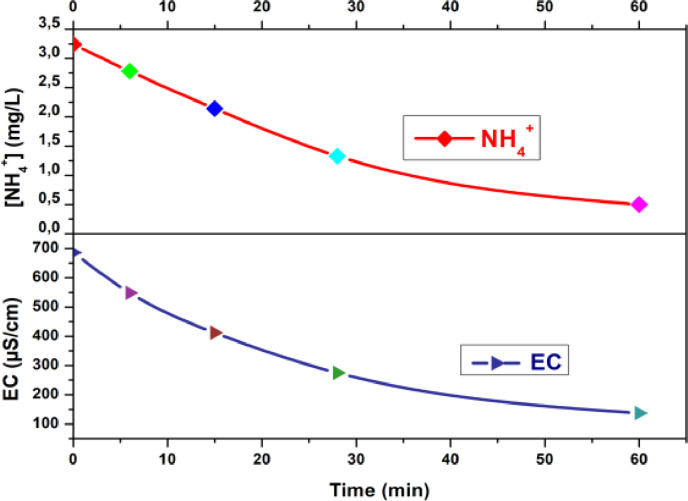
Ammonium concentration and EC in dilute vs time.

Figure 3 show respectively the variation of NH_4_^+^ concentration and EC in dilute as function of time.

Both parameters exhibit a progressive decrease over time, converging to NH_4_^+^ concentration of 0.5 mg/L and EC of 137.2 μS/cm at the 60‐minute mark. At 28 minutes, NH_4_^+^ concentration reaches 1.33 mg/L, corresponding to a NH_4_^+^ removal rate of 58.91 %. However, achieving a NH_4_^+^ concentration of 0.5 mg/L, indicating an 84.60 % NH_4_^+^ removal, requires a total treatment time of 60 minutes, an additional 30 minutes compared to the previous milestone. This discrepancy can be attributed to several factors:

- Ionic competition between NH_4_^+^ and other cations in the water, driven by their concentrations, higher valences (e. g., Ca^2+^ and Mg^2+^), ionic mobility, hydration energy, and hydration size.[^17^, ^26^, ^27^] Conversely, Yang et al^[14]^ suggest that NH_4_^+^ tetrahedral geometry confers it with high selectivity against spherical cations (e. g., Mg^2+^ and Ca^2+^).
- The similarity in polarity and hydraulic radius between NH_4_^+^ and water molecules.^[28]^
- The chemical composition of the CMX membrane, featuring fixed sulfonic groups and exhibiting non‐selective permeability to all ions, from highly charged to less charged species.^[29]^
- The inverse relationship between solution EC and ion removal duration, with lower EC solutions requiring more time for ion removal, resulting in a lower ammonium removal rate.

NH_4_^+^ removal is observed to increase as competitive cations are depleted from the water over time. These findings suggest potential enhancements in NH_4_^+^ removal through optimization of electrodialysis (ED) operating conditions. Future research should explore optimal ED parameters, such as applied voltage, current intensity, flow rate, and choice of ion exchange membrane pair, to further improve NH_4_^+^ removal efficiency.

**Figure 4:**
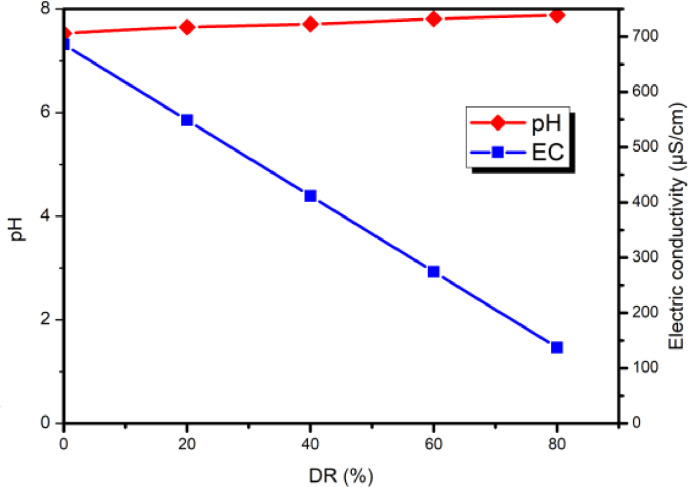
Variation of pH and EC in dilute as a function of demineralization rate.

Variations in EC and pH as a function of DR for dilute water are shown in Figure 4.

EC decreases linearly with the DR, which is explained by the fact that the EC of a solution is affected by the equivalent concentration and equivalent conductivity of the ions present in the solution.[^17^, ^30^] As the ions are removed, the conductivity of the solution decreases and the DR increases. As for the pH, it increases slightly with the DR from an initial value of 7.53 to a final value of 7.88. Variations in pH can affect the effectiveness of ED, but the impact is less significant.^[31]^ The pH determines the calco‐carbonic balance of water, its variation depends on the ionic strength, alkalinity and CO_2_ concentration, according to the equation (3).^[32]^ 3

(3) $$pH=\;{pK}_{1}-\epsilon \;+\log\left[{HCO}_{3}^{-}\right]\;-\log\left[{CO}_{2}\right]$$

Where ϵ is expressed as a function of the ionic strength μ of the solution $\epsilon =\frac{\sqrt{\mu }}{1+\sqrt{\mu }}$ and pK_1_ the carbonic acid acidity constant.

**Figure 5:**
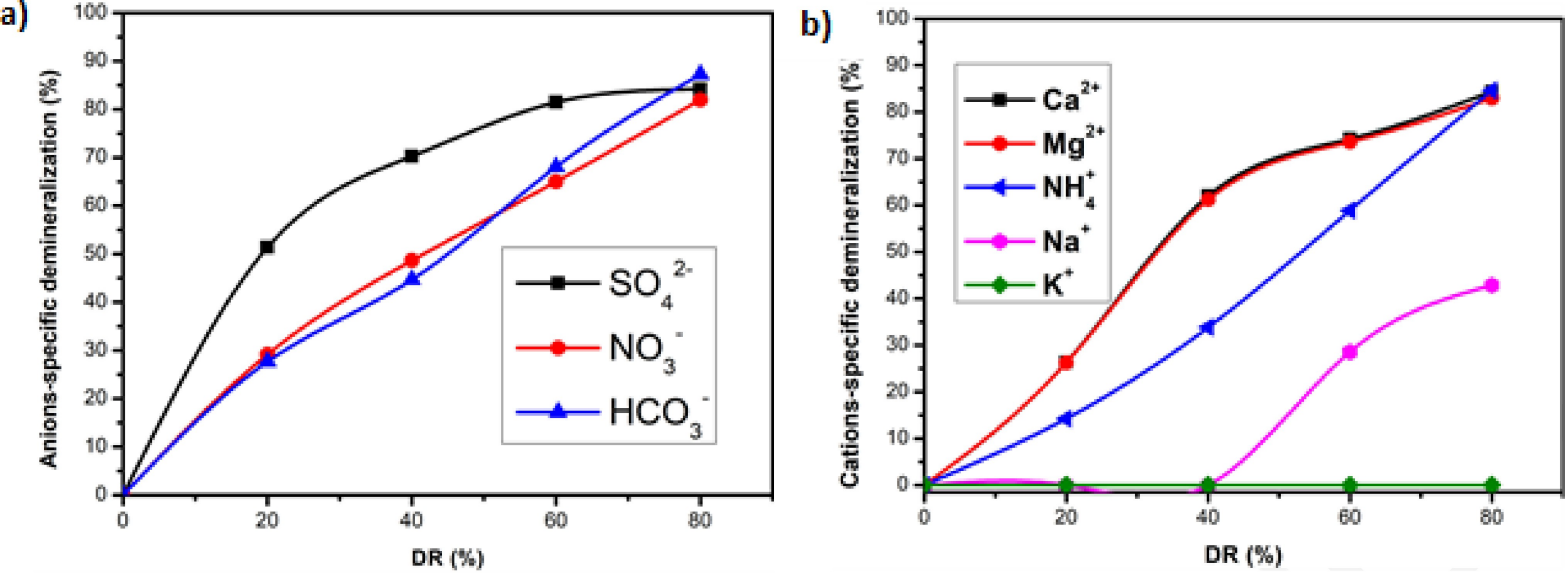
Selectivity of AXE and CMX membranes. a) SD of anions as a function of demineralization rate. b) SD of cations as a function of demineralization rate.

To investigate the selectivity of AXE and CMX membranes, Figure 5 gives respectively the SD of anions for AXE **(**figure 5a **)** and the SD of cations for CMX (Figure 5b).

Figure 5a shows the variation of the specific demineralization (SD) of the anions (SO_4_^2−^, NO_3_^−^ and HCO_3_^−^) as a function of the demineralization rate (DR). The SD of each anion increases in proportion to the DR, reaching 84.16 % removal for SO_4_^2−^, 87.23 % for HCO_3_^−^, and 81.98 % for NO_3_^−^ when the DR reaches 80 %. In particular, SO_4_^2−^ is eliminated first and quickly when the DR is between 0 and 40 %, then stabilizes from 60 % DR. This behavior can be attributed to the AXE membrane used, which is a non‐selective membrane allowing all ions to pass through, from the most charged such as SO_4_^2−^ to the least charged.^[24]^ In addition, SO_4_^2−^ ions have a high hydration energy and are preferentially exchangeable in AEMs due to their higher valence.^[33]^ This is in line with the results of Tahaikt et al^[24]^ who studied sulfate removal with two anion exchange membranes AXE and ACS and concluded that sulfate removal was greater with the AXE membrane. For NO_3_^−^ and HCO_3_^−^, removal rates are initially similar up to 20 % DR, after which HCO_3_^−^ removal decreases between 20 % and 50 % DR before increasing again between 60 % and 80 % DR. This trend can be attributed to the lower hydration energy and higher ionic mobility of NO_3_‐ compared to HCO_3_^−^.^[23]^ Despite the higher concentration of HCO_3_^−^ in the feed water, its removal only increases as NO_3_^−^ concentrations decrease in dilute water. The transport order of ions is as follows: SO_4_^2−^>NO_3_^−^>HCO_3_^−^. On the other hand, ion transport through an IEMs is influenced by thermodynamic and kinetic factors that could explain why one ion is eliminated before another. These factors affected by the distinct physicochemical characteristics of the ions, including their ionic radius, charge density, hydrated radius and hydration free energy.^[33]^ Mubita et al^[34]^ pointed out that ED membrane selectivity between anions can be improved by increasing the ion affinity difference, increasing the AEMs thickness and decreasing the AEMs charge density.

Figure 5b depicts the variation of specific demineralization (SD) of cations (NH_4_^+^, Ca^2+^, Mg^2+^, Na^+^ and K^+^) with demineralization rate (DR). The removal of divalent cations Ca^2+^ and Mg^2+^ increases proportionally with DR, reaching 82.84 % for Ca2+ and 82.64 % for Mg^2+^ at a DR of 80 %. NH_4_^+^ removal progressively increases with DR, reaching 84.60 % at a DR of 80 %. Na^+^ removal commences at a DR of 40 %, following the removal of Ca^2+^ and Mg^2+^, achieving only 42.45 % removal at a DR of 80 %. Due to its low concentration in water and small hydrated radius, K^+^ is not removed. The order of ion transport is as follows: Ca^2+^>Mg^2+^>NH^4+^>Na^+^>K^+^. The CMX membrane is a non‐selective membrane that allows all cations to pass through, from the most charged ions to the least charged ions.^[35]^ Despite the higher initial concentration of Na^+^ compared to NH_4_^+^ in water, NH_4_^+^ is removed prior to Na^+^. This phenomenon is attributed to the smaller hydration radius, lower hydration energy, and higher ionic mobility of NH_4_^+^ across the CMX membrane compared to Na^+^ (Table 2).[^17^, ^36^, ^37^]

**Table 2:** Properties of ions in aqueous solution at 25 °C.

Ion	Valence	Hydrated radii (nm)^[38]^	Hydration energy (kJ/mol)^[39]^
Mg^2+^	2	0.428	−1830
Ca^2+^	2	0.412	−1505
Na^+^	1	0.358	−365
K^+^	1	0.331	−295
NH_4_^+^	1	0.331	−285
SO_4_^2−^	2	0.378	−1080
NO_3_^−^	1	0.335	−300
HCO_3_^−^	1	–	−335

In Additionally, Aliaskari and Schäfer^[40]^ point out that the quality of the feed water, including contaminant concentration, pH and salinity, significantly influences contaminant removal during the ED process. For example, several studies have shown that nitrate removal is particularly high in the presence of NaCl, which can be explained by the high diffusion and ionic mobility of nitrates, as well as their competitive removal with other anions such as chloride, bicarbonate and fluoride.[^25^, ^41^, ^42^] Furthermore, the removal of arsenic(V) by ED in brackish water is relatively low due to the ionic characteristics of the other anions present, particularly chloride.^[13]^ Conversely, various studies have reported very high fluoride removal rates (up to 99 %) in brackish water in the presence of other ions.[^43^, ^44^, ^45^] However, research indicates that anions with higher mobility and diffusivity, such as chloride, nitrate and hydroxide, compete with fluoride and hinder its removal, thus prolonging the treatment time of the ED process.^[40]^

**Figure 6:**
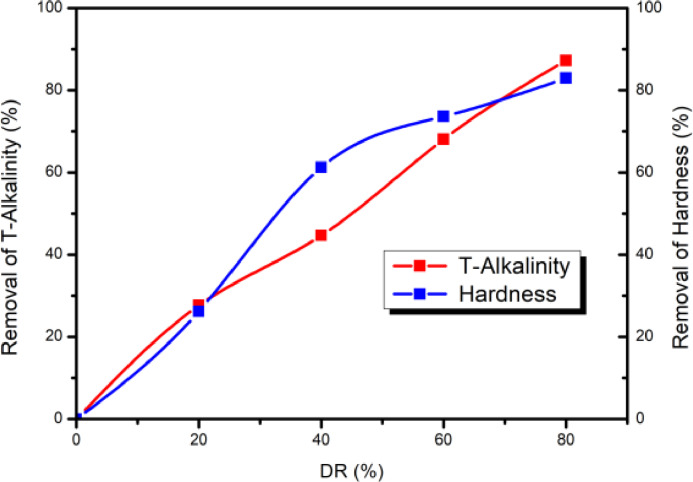
Removal of T‐Alkalinity and hardness as a function of demineralization rate.

Figure 6 shows the variation of removal of hardness and T‐Alkalinity as a function of DR.

Hardness and T‐Alkalinity removal increases with the demineralization rate (DR), reaching 87.23 % and 82.93 % respectively at a DR of 80 %. Furthermore, the removal of hardness and T‐Alkalinity has an impact on the calco‐carbonic balance of the treated water, as this balance depends on the concentrations of Ca^2+^ and HCO_3_^−^. These ions represent the water‘s hardness and T‐Alkalinity respectively. Consequently, when Ca^2+^ concentration decreases, hardness decreases, and when HCO_3_^−^ concentration decreases, alkalinity decreases. It is therefore essential to assess the quality of the treated water obtained.

### Statistics Analysis

Descriptive statistics play a crucial role in the synthesis and initial understanding of data, providing an essential basis for more advanced statistical analyzes and informed decision‐making in various fields Table 3.

**Table 3:** Descriptive statistics.

	Units	Mean	Std. Deviation	Minimum	Maximum
DR	%	40.00	31.62	0.00	80.00
EC	μS/cm	411.60	216.93	137.20	686.00
T	°C	23.56	3.29	19.60	28.30
pH	–	7.72	0.12	7.53	7.88
NH_4_^+^	mg/L	2.01	1.11	0.50	3.25
SO_4_^2−^	mg/L	15.52	12.63	5.77	36.48
NO_3_^−^	mg/L	12.38	7.16	4.05	22.49
T‐Alkalinity	meq/L	2.56	1.60	0.60	4.70
P‐Alkalinity	meq/L	0	0	0	0
HCO_3_^−^	mg/L	156.10	97.62	36.59	286.59
Hardness	meq/L	3.01	2.04	1.00	5.88
Ca^2+^	mg/L	52.91	35.89	17.63	103.33
Mg^2+^	mg/L	4.46	3.02	1.48	8.70
K^+^	mg/L	2.06	0.00	2.06	2.06
Na+	mg/L	91.05	21.46	60.70	106.22
Current Intensity	A	0.16	0.03	0.13	0.19

**Figure 7:**
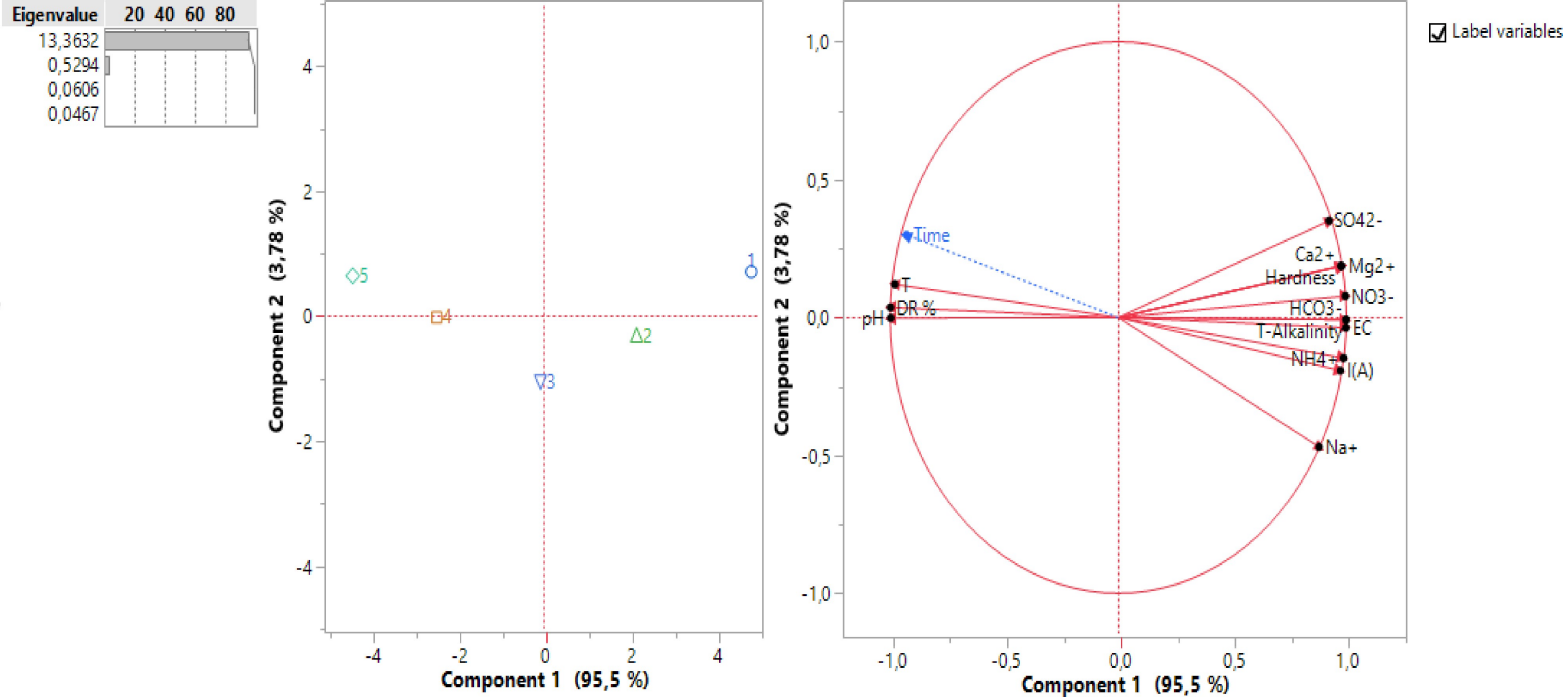
Correlation circle of variables (C1×C2 at 99.28 %).

Figure 7 shows the correlation circle characterized by a distribution of variables along the two main axes C1 and C2; on the C1 axis, a gradient of decreasing concentration is observed on the right‐hand side of the graph, showing high concentrations of the physico‐chemical parameters studied: SO_4_^2−^, HCO_3_^−^, NO_3_^−^, NH_4_^+^, Na^+^, Mg^2+^, Ca^2+^, EC, T‐Alkalinity and Hardness, which are positively correlated on the C1 axis with time (T_0_=0 at high concentrations). During the ED process, their concentrations will decrease towards the left of this graph at the time (T_5_=60 minutes) when the demineralization rate of DR reaches its maximum.

PCA explores the distribution of variable concentrations and their correlations during the ED process, from input to output, marking the best performance.

**Figure 8:**
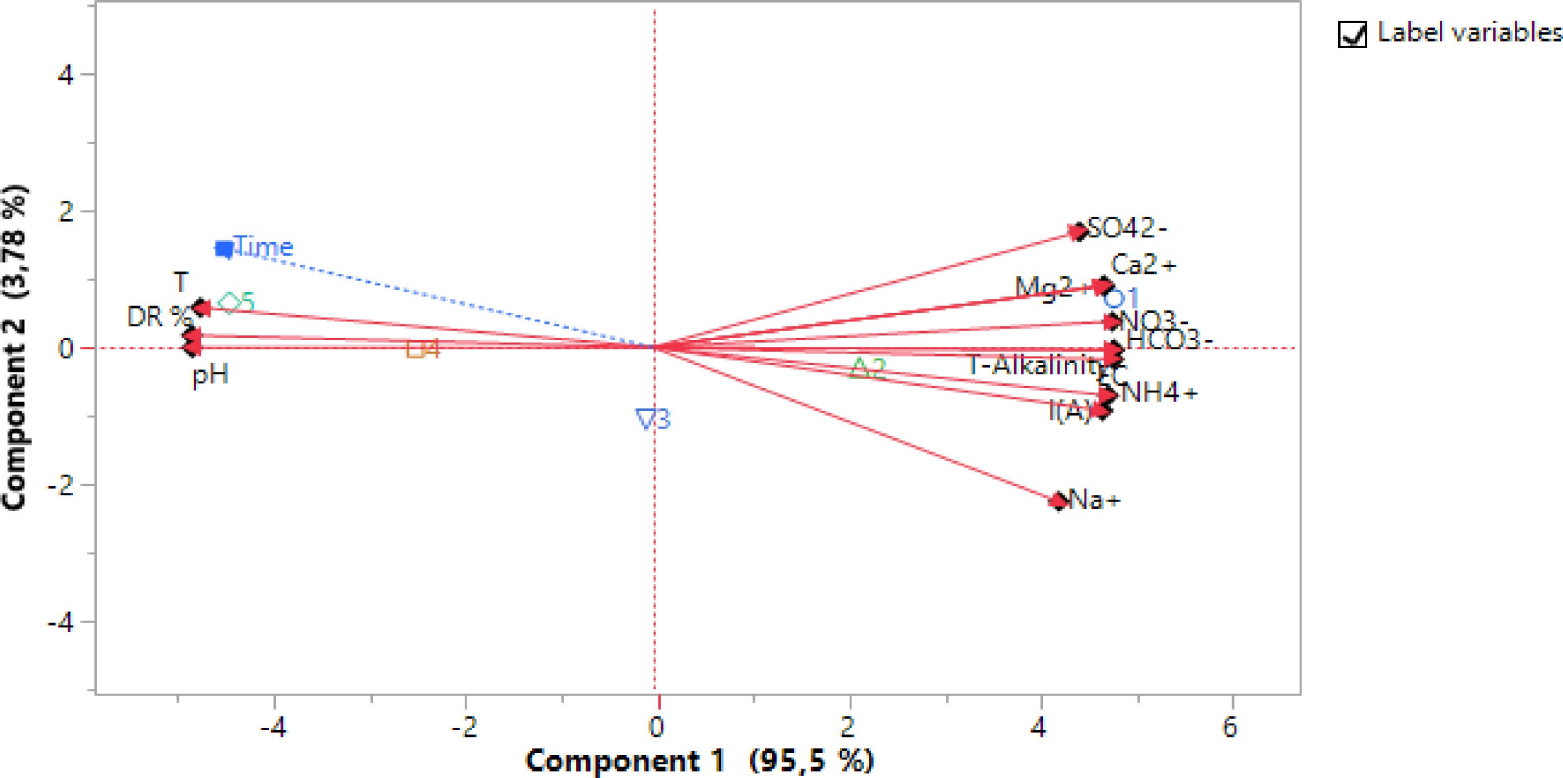
Biplot projection of variables and individuals with an inertia of (C1×C2 at 99.28 %).

Figure 8 shows the Biplot projection of variables and individuals along the C1 and C2 axes.

Over time, we observe very high raw concentrations at time t_1_=0 min, and at t_2_, t_3_, t_4_ up to t_5_=60 minutes, the concentrations of the physico‐chemical parameters indicated on the right of the graph progressively decrease, as shown by the performance of each treated element. From the right to the left of the graph, we observe a decreasing concentration gradient, which explains the good performance of the ED process used, increasing the DR with time (t_1_ to t_5_). Points 1 to 5 represent lines considered as individuals characterizing the concentration gradient.

### Water Quality

**Figure 9:**
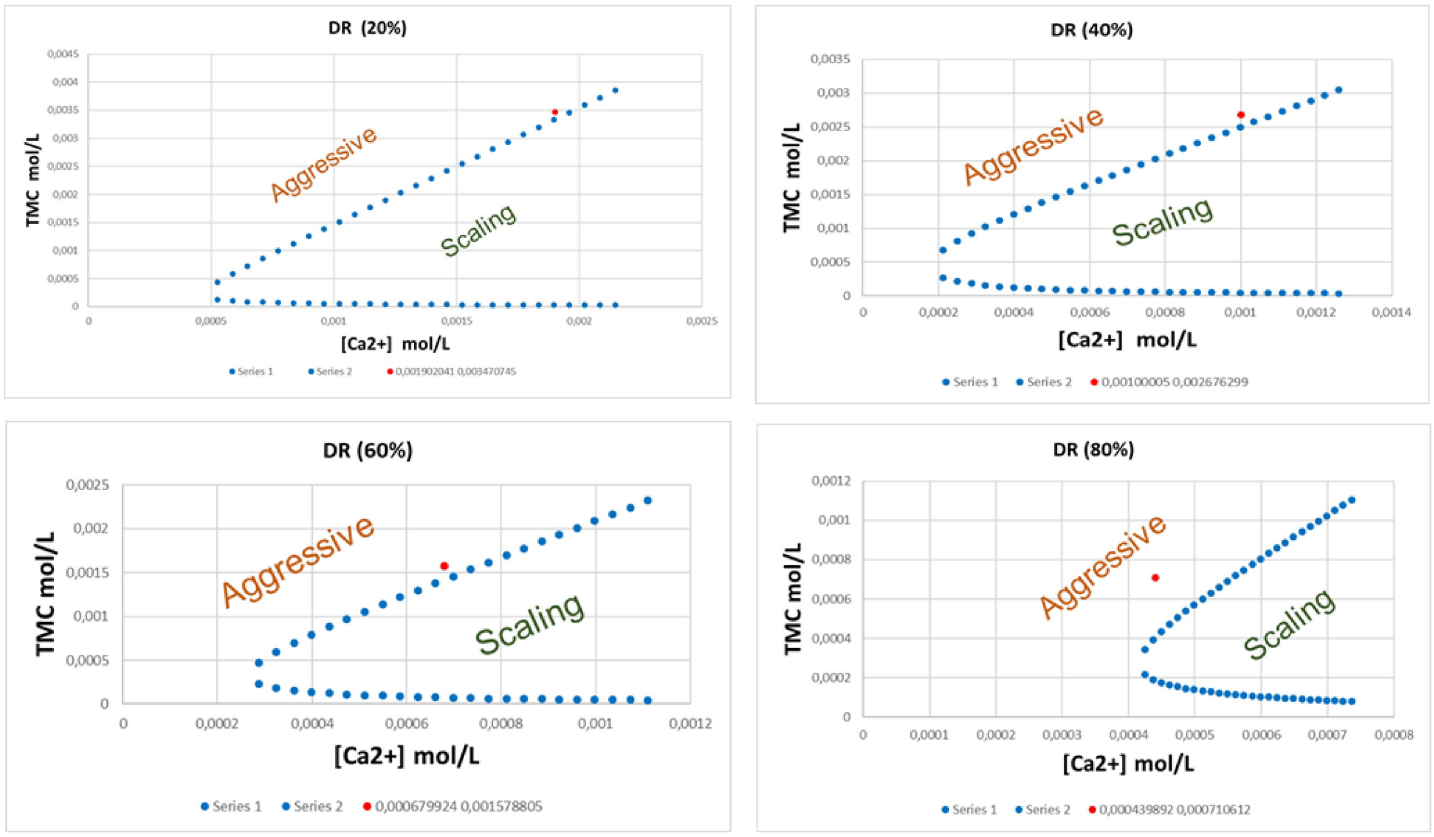
Calco‐carbonic equilibrium curves of dilute solutions at different DR (20, 40, 60 and 80 %).

After the characterization of the dilute solutions obtained by ED, the quality of the water is evaluated based on calco‐carbonic equilibrium study according to the method of Legrand‐Poirier for all the demineralization rate. The equilibrium curves are plotted in Figure 9 and the dilute obtained for all DR (20 %, 40 %, 60 % and 80 %) are located (red points) according to the calco‐carbonic equilibrium curves. It appears that all the dilute solutions obtained by ED are located in the aggressive zone of the curve and the higher the DR, the more aggressive the water. At DR of 20 %, the point is located in the aggressive zone close to the equilibrium curve, which means that a small amount of reagent will be sufficient to restore the water‘s equilibrium. Conversely, at DR of 80 %, the point moves away more and more from the equilibrium curve and consequently, the requested amount of reagent will be significant.

Then, using a calculation program, the amount of calcium to be added for each DR is calculated for all samples to reach the equilibrium curve. Table 4 gives the amounts of Ca^2+^ to be added as well as the water evaluation after re‐balancing.

**Table 4:** Evaluation of water quality after Ca^2+^ addition.

Characteristics	DR=20 %	DR=40 %	DR=60 %	DR=80 %
Initial [Ca^2+^] (mg/L)	76.23	40.08	27.25	17.63
Legrand‐Poirier	Slightly aggressive	Aggressive	Aggressive	Aggressive
[Ca^2+^] to add (mg/L)	2.80	3.00	3.16	4.50
Evaluation	Balanced	Balanced	Balanced	Balanced

## Conclusions

This study evaluates the feasibility of ammonium removal by electrodialysis (ED) using real groundwater solutions from the city of Kenitra in Morocco, initially spiked with 3 mg/L NH_4_Cl. The results demonstrate efficient ammonium removal, reaching a final concentration of 0.5 mg/L corresponding to a demineralization rate (DR) of 80 % after 60 minutes of ED treatment. Since the cation exchange membrane used lacks selectivity for ammonium, ED treatment also results in the removal of other cations present in the diluted water, albeit to varying degrees. Similarly, anions are removed depending on the anion exchange membrane used. ED‐treated water complies with Moroccan standards for ammonium content. However, the Legrand‐Poirier method, used to assess water quality after ED treatment compared with DR, indicates aggressive water characteristics. Consequently, the addition of calcium is deemed necessary to balance the water and make it fit for human consumption. These preliminary results are promising and suggest that ED could serve as an effective and environmentally‐friendly technology for removing ammonium from drinking water. This approach could mitigate the health risks associated with the consumption of ammonium‐contaminated water, while promoting sustainable management of water resources. Future efforts will focus on optimizing the ED operating parameters specifically for ammonium removal, and carrying out further assessments on groundwater naturally polluted by ammonium. With regard to the disposal of ammonium concentrate, and in line with zero liquid discharge (ZLD) targets, the preferred strategy is to explore its reuse potential for fertigation in agriculture.

## Supporting Information

The supporting information contains the characteristics of the TS‐2‐10 ED pilot plant used in this study as well as the properties of the ion exchange membranes used.[^22^, ^23^]

## Conflict of interests

The authors declare no conflict of interest.

1

## Supporting information

As a service to our authors and readers, this journal provides supporting information supplied by the authors. Such materials are peer reviewed and may be re‐organized for online delivery, but are not copy‐edited or typeset. Technical support issues arising from supporting information (other than missing files) should be addressed to the authors.

Supporting Information

## Data Availability

The data that support the findings of this study are available from the corresponding author upon reasonable request.
