# Supplementary material for: Evaluation Study of Ammonium Removal from Groundwater by Electrodialysis: Case Study of Real Groundwater from the City of Kenitra in Morocco
Source: ChemistryOpen. 2024 Apr 9;13(5):e202300163. doi: 10.1002/open.202300163 (PMC11095167; doi:10.1002/open.202300163)
Supplement: Supplementary file 1 — Supporting Information [file OPEN-13-e202300163-s001.pdf]

ChemistryOpen

Supporting Information

Evaluation Study of Ammonium Removal from Groundwater by Electrodialysis: Case Study of Real Groundwater from the City of Kenitra in Morocco

Mohamed Hazra,* Mohamed Ouzbair, Ibrahim Maolida, Omar Elrhaouat, Mustapha Tahaikt, Azzedine Elmidaoui, Mohamed Taky, and Sakina Belhamidi*

Table contents

Experimental Section 1
Description of the electrodialysis pilot plant 1

The additional information cited in this document completes the experimental section by describing the electrodialysis pilot used, and providing information on the ion exchange membranes used in our work.

Experimental Section

Description of the electrodialysis pilot plant

The TS-2-10 electrodialysis pilot plant used is the same as described in other works.[1,2] **Figure S1** shows some photos of the TS-2-10 Pilot. The characteristics of the pilot plant are grouped in **Table S1**.[1] The properties of the AXE anion and CMX cation exchange membranes used are given in **Table S2**.[2]

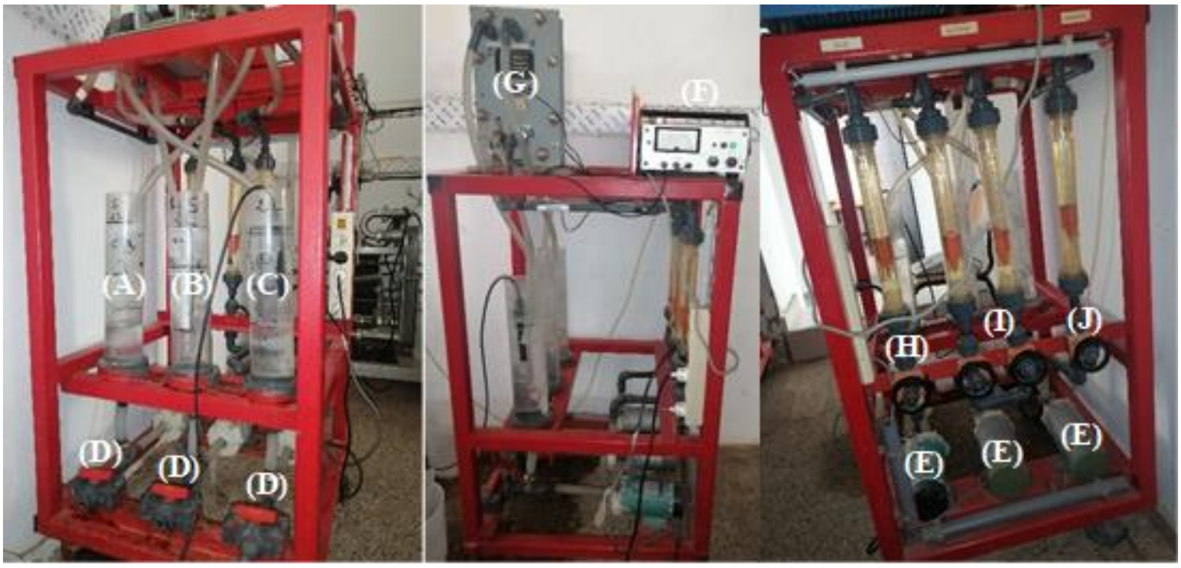

Figure S1. TS-2-10 pilot. **(A)**: Concentrate, **(B)**: Electrode rinse, **(C)**: Dilute, **(D)**: Flow control valves, **(E)**: Pumps, **(F)**: DC power supply, **(G)**: Membrane stack, **(H)**: Flow dilute, **(I)**: Flow electrode, **(J)**: Flow concentrate.

Table S1. Characteristics of the TS-2-10 pilot equipment.

**Description of equipment**	**Pilot TS-2-10**
Membrane active area, (cm^2)	200
Number of cell pair	10
Separator frame gasket	EPDM
Separator and distributor	EP+PP
Electrode	
Anode	Platinium-coated titanium
Cathode	Platinium-coated titanium
Parameters of functioning	
• Flow dilute compartment (L/h)	180
• Volume dilute compartment (L)	2

• Flow concentrate compartment (L/h)	180
• Volume compartment (L)	2
Electrodes compartments	
• Flow (L/h)	150
• Volume (L)	2
Current max, A	9
Voltage max, V/cell	1

Table S2. Properties of AXE and CMX membranes.

**Type of membrane**	**Thickness, mm**	**Active area, cm^2**	**Electrical resistance, $Ohm.cm^2$**	**Exchange capacity, $meq.g^{-1}$**
AXE	0.17	200	1.4	2.5-3.5
CMX	0.17	200	2.39	1.50

[1] A. Elmidaoui, F. Elhannouni, M. A. M. Sahli, L. Chay, H. Elabbassi, M. Hafsi, D. Largeteau, *Desalination* **2001**, *136*, 325.

[2] F. Z. Addar, I. Mohamed, S. Kitanou, M. Tahaikt, A. Elmidaoui, M. Taky, *Water Science and Technology* **2023**, wst2023423.
